# Gender dysphoria in twins: a register-based population study

**DOI:** 10.1038/s41598-022-17749-0

**Published:** 2022-08-04

**Authors:** Georgios Karamanis, Maria Karalexi, Richard White, Thomas Frisell, Johan Isaksson, Alkistis Skalkidou, Fotios C. Papadopoulos

**Affiliations:** 1 Department of Medical Sciences, Psychiatry, Uppsala University, Uppsala, Sweden; 2 Norwegian Institute of Public Health, Oslo, Norway; 3 Clinical Epidemiology Division, Department of Medicine Solna, Karolinska Institute, Stockholm, Sweden; 4 Department of Women’s and Children’s Health, Center of Neurodevelopmental Disorders (KIND), Centre for Psychiatry Research, Karolinska Institutet, Stockholm Health Care Services, Region Stockholm, Stockholm, Sweden; 5 Department of Women’s and Children’s Health, Obstetrics and Gynecology, Uppsala University, Uppsala, Sweden

**Keywords:** Medical research, Epidemiology

## Abstract

Both genetic and environmental influences have been proposed to contribute to the variance of gender identity and development of gender dysphoria (GD), but the magnitude of the effect of each component remains unclear. We aimed to examine the prevalence of GD among twins and non-twin siblings of individuals with GD, using data derived from a large register-based population in Sweden over the period 2001–2016. Register data was collected from the Statistics Sweden and the National Board of Health and Welfare. The outcome of interest was defined as at least four diagnoses of GD or at least one diagnosis followed by gender-affirming treatment. A total of 2592 full siblings to GD cases were registered, of which 67 were twins; age at first GD diagnosis for the probands ranged from 11.2 to 64.2 years. No same-sex twins that both presented with GD were identified during the study period. The proportion of different-sex twins both presenting with GD (37%) was higher than that in same-sex twins (0%, Fisher’s exact test *p*-value < 0.001) and in non-twin sibling pairs (0.16%). The present findings suggest that familial factors, mainly confined to shared environmental influences during the intrauterine period, seem to contribute to the development of GD.

## Introduction

Gender identity is defined by the American Psychological Association as a person’s deeply‐felt, inherent sense of being male, female or alternative gender, such as gender queer, gender non-conforming, gender neutral, that may or may not correspond to a person’s sex assigned at birth or to a person’s primary or secondary sex characteristics^1^. Gender incongruence is a condition defined in the 11th Revision of the International Statistical Classification of Diseases and Related Health Problems (ICD-11) as “a pronounced, persistent incongruence between the individual’s experience of gender and the sex assigned”, a change from the category Gender Identity Disorder, used in ICD-10^2,3^. When this mismatch causes discomfort or distress, it is referred to as gender dysphoria (GD), according to the fifth Edition of the Diagnostic and Statistical Manual of Mental Disorders (DSM-5)^4^. During the last decades, an increase in the prevalence of GD has been reported^5^ and multiple studies have shown that people with GD have a higher risk for, among others, depression, anxiety disorders, suicide, and less access to health care facilities^6^. The etiology of gender incongruence and GD remains mostly unknown. In recent years, there has been mounting evidence on biological, more specifically genetic, factors, in addition to psychosocial factors that may underlie the etiology of GD.

The twin study design is a valuable tool for the examination of genetic and environmental influences on physical and behavioral traits. The premise of the twin study is that monozygotic twins (MZ) share the same genotype, whereas dizygotic twins (DZ) share an average of 50% of their genes and are therefore genetically as similar as non-twin siblings. According to the equal environments assumption, both types of twins are equally exposed to environmental factors during upbringing. Twin studies that compare concordance rates for MZ twins to those for DZ twins can therefore provide estimates of heritability, i.e., the degree to which genetic factors as well as shared and non-shared environmental factors contribute to the etiology of a phenotype. The role of genetic factors in the development of GD remains unclear, despite several previous twin studies in the last two decades^7–15^. These previous studies were all limited by small samples and unclear generalizability due to the selection of participants, and that most of them focused on gender nonconformity and measures of gender identity rather than GD.

Gender nonconformity in childhood has been examined by assessing phenotypes such as gender role, expression or behavior, with sometimes non-validated instruments^8,9^. Gender nonconformity has been shown to be a predictor of sexuality rather than gender identity and gender incongruence later in life^7,8^. Even though gender identity and gender incongruence are more similar to the construct of GD, they are far from the same, as most people with gender incongruence do not develop GD defined as a term in clinical settings. The few twin studies that have examined outcomes related to GD have shown a possible genetic influence, but with varying heritability or concordance rates. Coolidge et al. estimated heritability at 62%^10^, while Sasaki et al. found a heritability of 41% for adolescent assigned females (aF) and 11% for adult aF, and no genetic effects for assigned males (aM)^14^. Heylens et al. reported a concordance of 39.1% in MZ twins and 0% in DZ pairs^12^. Diamond found that the concordance among same-sex DZ pairs was 33% for aM and 23% for aF^11^.

In addition, these studies have some methodological limitations, such as small sample size^12,14^, heterogeneous population^11^ and parent rating rather than self-rating^10^.

Beyond twin studies, a study has also reported familial cases of GD in 5 non-twin sibling or father-child pairs^16^. Likewise, a Spanish study on 995 consecutive transsexual probands reported 12 pairs of GD in non-twin siblings^17^. This study further showed that the probability that a sibling of a transsexual will also be transsexual was 4.48 times higher for siblings of male-to-female than for siblings of female-to-male transsexual probands, and 3.88 times higher for the brothers than for the sisters of transsexual probands.

Overall, evidence for a genetic etiology of GD remains precarious and is mainly compromised by the large heterogeneity in study design, outcome definitions, measures of assessing GD and the assessment of potential confounders across different studies. In the present study, we aimed to examine the prevalence of GD among same- and different-sex twins compared to a non-twin sibling cohort derived from a large register-based population in Sweden over the period 2001–2016.

## Methods

### Participants and procedure

Our source population consisted of all individuals who had received a GD diagnosis according to the 10th version of the International Classification of Diseases (ICD-10) between January 1, 2001, and December 31, 2016, and were at least 10 years old (N = 4374). Individuals who had also been diagnosed with the previous ICD-8 and ICD-9 GD diagnoses (N = 100) or had previously changed legal sex (N = 22) were excluded, given that these were not incident cases. Using the National Patient Register (NPR) and Prescribed Drugs Register (PDR), individuals with surgical or prescribed hormonal gender-affirming treatment prior to first diagnosis were also excluded (N = 166), in order to achieve higher validity of our definition, as some components of gender-affirming treatment could theoretically be given for indications other than gender dysphoria. The decision to have 2001 as the starting year of the follow-up period was partly because of the introduction of ICD-10 in Sweden in 1997 and partly because the outpatient NPR started in 2001. The study was designed in 2016 and that year was, therefore, the end of the follow-up period.

Gender-affirming interventions consisted of hormonal and surgical treatments. Gender-affirming hormonal treatments, including puberty blockers, were identified by ATC codes in the PDR and gender-affirming surgical treatments were identified by NOMESCO Classification of Surgical Procedures (NCSP) codes in the NPR^18^. The following codes were used: G03B (testosterone), G03C, L02AA, G03D, L02AB, G03H, L02BB, G04CB, C03DA01, L02AE and H01CA (antiandrogens and estrogen), L02AE and H01CA (puberty blockers), HAC10, HAC15, HAC20, HAC99, HAD20, HAD30, HAD35, HAD99 and HAE99 (mastectomy and breast reductions), HAD00, HAD10, HAD99, HAE00, HAE20 and HAE99 (breast reconstruction), KFH50, KGV30, KGW96, KGH96, LCD00, LCD01, LCD04, LCD10, LCD11, LCD96, LCD97, LED00, LEE10, LEE40, LEE96, LFE10, LFE96 and KGC10 (genital surgeries), DQD40 (larynx surgery).

Individuals with GD were defined as those who received one GD diagnosis (F64.0 Transsexualism, F64.8 Other gender identity disorders, or F64.9 Gender identity disorder, unspecified) in the NPR on at least four occasions, or those who received at least one GD diagnosis followed by gender-affirming treatment. The first definition was utilized in order to include individuals with clinically significant GD who, for a variety of reasons, did not receive gender-affirming treatment, while the latter was used in order not to miss individuals with treated GD but with too short follow-up time to obtain four diagnoses. The definition, as proposed by Indremo et al.^18^, showed to be indicative of clinically relevant GD in the Swedish setting when compared with other register definitions.

**Figure 1:**
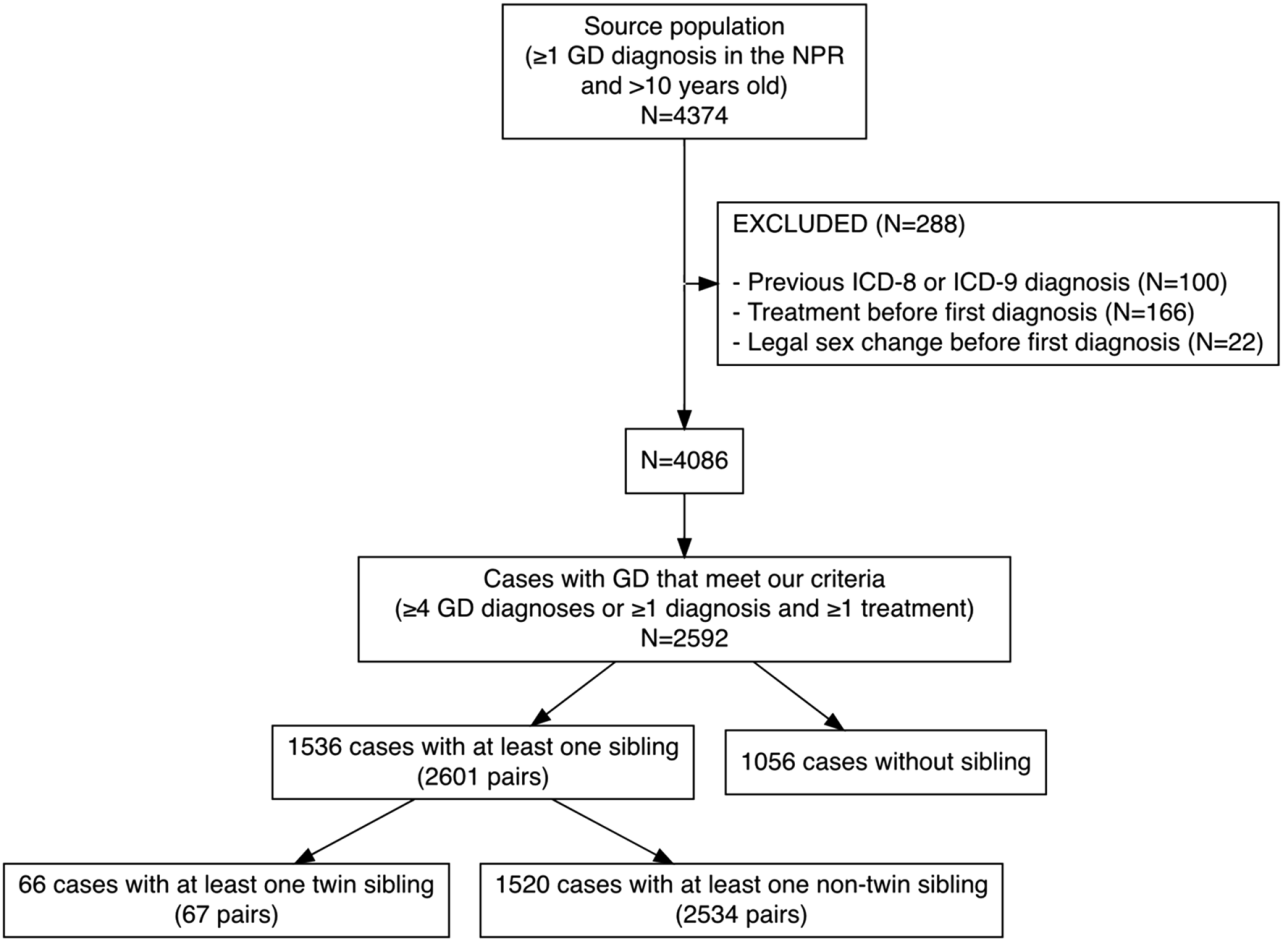
Flow chart of study population selection.

We identified 2592 individuals with GD according to our definition, of which 1536 had at least one full sibling, which yielded 2601 siblings (Fig. 1). Twins were defined as full siblings born in the same year and month as the proband. Both twin and non-twin pairs are not unique given that a pair of two siblings (e.g., A and B) would appear twice, if they both have a GD diagnosis, because each individual would be proband for its sibling (A–B and B–A).

Register data was collected from the Statistics Sweden (SCB) and the National Board of Health and Welfare. All registers in Sweden use the 10-digit National Registration Number, a unique personal identifier assigned to all Swedish residents to allow linkage between registers. When retrieving data from the registers, all personal identifiers are replaced with consecutive numbers to secure anonymity. From the National Board of Health and Welfare, we retrieved data from the NPR, comprising information on primary and secondary ICD diagnoses from visits to specialist outpatient care since 2001 and inpatient health care since 1964, including surgeries, admission, and discharge dates. The inpatient register has nation-wide coverage since 1987, while the outpatient register started with a coverage between 8 and 18% during its first three years and reached 93% coverage by 2015. Sociodemographic data on birth dates, assigned sex at birth, change of legal sex, country of birth, and education level were retrieved from the Swedish Total Populations Register and linked to the NPR and PDR, that includes data on all redeemed medication since July 1, 2005. Data available from the Swedish Multi-Generation register allow the linkage of individuals born in 1932 and later, to parents, siblings and offspring^19^. The informed consent was waived by the Central Ethical Review Board in Stockholm and all methods were performed in accordance with the relevant guidelines and regulations. The study was approved by the Central Ethical Review Board in Stockholm (Dnr. Ö30-2016).

### Statistical analysis

The percentage of probands with a twin or non-twin sibling with GD, totally and by assigned sex in birth, was calculated along with descriptive statistics of the population. Fisher’s exact test and Mann–Whitney U test were used, and a p-value of 0.05 was set as the level of statistical significance. The statistical software environment R was used for data management and statistical analysis^20–22^ which were conducted in RStudio (R version 4.1.2 (2021-11-01), RStudio 2021.09.1 Build 372, tidyverse 1.3.1, data.table 1.14.2).

## Results

**Figure 2:**
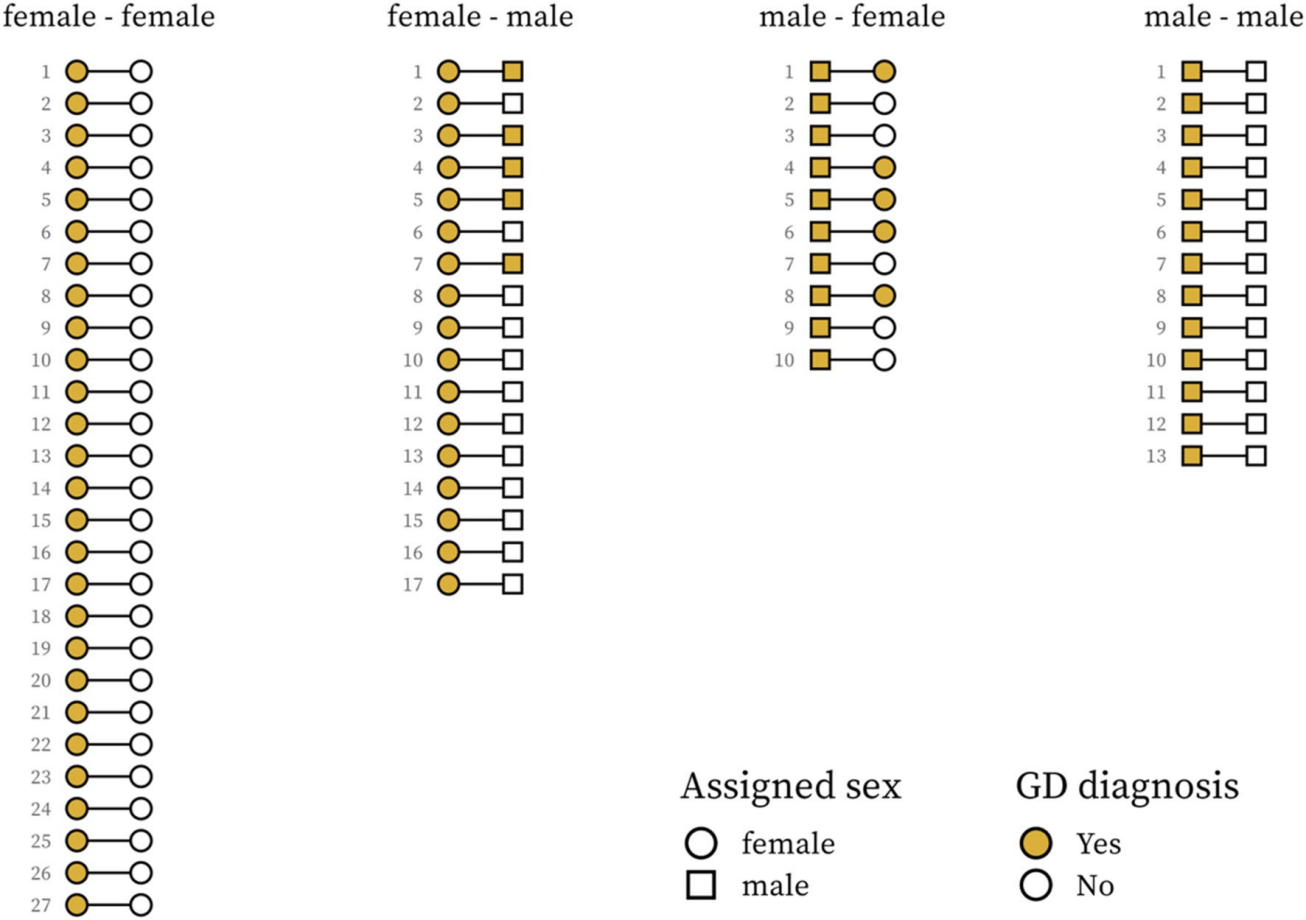
Gender dysphoria (GD) diagnosis among twins by proband and co-twin assigned sex.

Over the period 2001–2016, 2592 full siblings were registered, of which 67 were twins; one set of triplets (one proband with two “co-twins”) was also identified (Fig. 2), raising the number of twin pairs to 67 for the 66 probands.

The 66 probands were born between August 1950 and September 2003. Age at first GD diagnosis for the probands ranged from 11.2 to 64.2 years (Table 1). The non-twin sibling pairs consisted of 1520 unique probands and a total of 2534 full siblings.

**Table 1:** Characteristics of the study participants.

	Concordant twins (N = 10, not unique)	Discordant twins (N = 57)	Concordant non-twin sibling pairs (N = 4, not unique)	Discordant non-twin sibling pairs (N = 2530)
**Age at first diagnosis, years**				
Mean age of proband at first diagnosis (SD)	32.1 (14.8)	25.5 (10.8)	24.7 (4.7)	27.4 (11.6)
Mean age difference of first diagnosis between siblings	3.7	NA	6.9	NA
p-value (derived from Mann–Whitney U test)	1.0		1.0	

	Probands in twin pairs (N = 67)	Siblings in twin pairs (N = 67)	Probands in non-twin pairs (N = 2534)	Siblings in non-twin pairs (N = 2534)
**Education, N (%)**				
9 years or less	14 (21%)	11 (16%)	662 (25%)	421 (16%)
10–12 years	30 (45%)	33 (49%)	998 (38%)	1086 (42%)
13 years or more	19 (28%)	17 (25%)	837 (32%)	819 (31%)
Unknown	4 (6%)	6 (9%)	104 (4%)	275 (11%)
**Country of birth, N (%)**				
Sweden	59 (88%)	65 (97%)	2089 (82%)	2412 (95%)
Other Nordic countries	0 (0%)	0 (0%)	16 (1%)	28 (1%)
Outside the Nordic countries	2 (3%)	2 (3%)	102 (4%)	92 (4%)
Unknown	6 (9%)	0 (0%)	327 (13%)	2 (0%)

Among the 67 twin siblings of the probands, there were 10 (14.9%) with GD according to our definition. There were 40 probands with a same-sex twin and 27 probands with a different-sex twin. All 10 twin siblings with GD were twins to a different-sex proband, i.e., among the twins to a proband of the same assigned sex, none had GD (*p* < 0.001; Table 2). There were 4 full siblings with GD (0.16%) among the 2534 non-twin siblings. The mean follow-up time from the first GD diagnosis until the end of study period was 5.4 years, ranging from 1 to 14.8 years for the twins with GD.

**Table 2:** Gender dysphoria (GD) in twin and non-twin siblings to probands of same or different assigned sex.

	GD in both siblings (N, %)	GD only in proband (N, %)	*p-*value (derived from Fisher’s exact test)
Different-sex twins (N = 27)	10 (37.0%)	17 (63.0%)	< 0.001
Same-sex twins (N = 40)	0 (0%)	40 (100.0%)	
Different-sex siblings (N = 1223)	2 (0.2%)	1221 (99.8%)	1
Same-sex siblings (N = 1311)	2 (0.2%)	1309 (99.8%)	

## Discussion

Based on registry data from a large Swedish population-based cohort, we compared the prevalence of GD among twin and non-twin siblings to persons with our definition of GD during a period of 16 years (2001–2016). No same-sex twins (and thus no MZ) with GD were identified. The prevalence in different-sex twin pairs was much higher than the prevalence in same-sex twins, as well as than that in non-twin sibling pairs. The different-sex pairs can, by definition, be only DZ twins, whereas the same-sex twin pairs can be either MZ or DZ. Since there were no twin siblings with GD among the 40 same-sex twin pairs, we can conclude that there are no concordant MZ twins, irrespective of the total number of MZ twins. By contrast, among the different-sex (i.e., DZ) twins there were 10 (37%) with GD. Interestingly, there were only 4 full siblings with GD (0.16%) among the 2534 non-twin siblings, a striking difference in concordance compared to the different-sex twin pairs, considering that they are genetically similar. This difference cannot be explained by genetics or shared childhood environments, pointing to a possible effect of intrauterine exposures.

Both genetic, epigenetic and environmental factors have been proposed to account for the variance in gender identity^8^. However, the true magnitude of the effect of each component has not been clarified so far. Table 3 summarizes the literature findings on gender identity-related outcomes in twin and non-twin siblings. Some previous twin studies have reported higher concordance in MZ than in DZ twins^11,12^, while some studies have shown moderate genetic influences in gender variation^7,9^, and others have shown that heritability accounts for 11%-70% of gender variance^10,14,15^. The large variability in study design, study period, outcome definitions and age of the studied populations across the different study groups may also underlie the heterogeneous findings of previous studies, thus not allowing a direct comparison with the results presented herein.

**Table 3:** Previous studies examining gender identity-related outcomes in twin and non-twin siblings.

Study	Method	N	Outcome	Findings	Comments
Bailey et al.^8^	Heritability of childhood gender nonconformity and adult masculinity–femininity in twins	1341 assigned male (aM) and 2441 assigned female (aF) twin pairs	Childhood gender non-conformity; continuous gender identity	Heritability for aM 0.50 and for aF 0.37	Retroactive assessment in adults
Coolidge et al.^10^	Heritability of gender identity disorder (GID) in twins	157 twin pairs	Six GID items based on DSM-IV criteria	Heritability of clinically significant GID 0.62	Children and adolescents
Knafo et al.^13^	Heritability of atypical gender role development in twins	5799 twin pairs	Masculinity and femininity; 24 items of pre-school activities inventory	Group heritability for aM 0.26–0.27 and for aF 0.42–0.50	Children 3–4 years
van Beijsterveldt et al.^15^	Heritability of cross-gender behavior during childhood in twins	4530 aF twin pairs	Cross gender behavior & cross gender identity	Genetic factors accounted for 70% of cross-gender behavior	Children 7 and 10 years
Alanko et al.^7^	Heritability of cross-gender behavior during childhood in twins	3261 twins	Shortened version of the Recalled Childhood Gender Identity/Gender Role Questionnaire	Heritability estimates for childhood gender atypical behavior 51% for aF and 29% for aM	Adults, recalled childhood atypical gender behavior before the age of 12 years
Gómez-Gil et al.^17^	Concordance for GID in twin and non-twin siblings	995 patients	GID according to DSM-IV and ICD-10, assessed with semi-structured clinical interviews and case discussion	12 non-twin and 2 twin pairs concordant for GID	Patients from gender identity clinics
Burri et al.^9^	Heritability for childhood gender typicality in twins	4426 twins	Childhood gender typicality; Four items assessing childhood sex-typed behavior and gender identity	Heritability for childhood gender typicality was 32% and for adult gender identity 11%	Adults, same-sex pairs (aF)
Heylens et al.^12^	Concordance for GID in twins	51 twin pairs	GID as reported in previous case reports or own assessment	Concordance among monozygotic twins 39.1% and among same and different-sex DZ twins 0%	Previous case reports and own unpublished cases
Diamond^11^	Concordance for GID in twins	112 twin pairs	Transitioning from living in the gender assigned at birth to that of the opposite gender, self-reported or clinical confirmed	Concordance among same-sex DZ pairs 33% for aM and 23% for aF	Age > 10 years, combined from bibliography and survey
Sasaki et al.^14^	Heritability of GID in twins	1961 aM and 2333 aF twin pairs	GID trait score, assessed with questionnaire items, based on DSM-IV	Heritability among aF in adults was 11%	Children, adolescents, and adults

Our study had a different outcome than most other studies that reported on outcomes related to gender identity, gender role or cross-gender behavior, i.e., traits and not clinical diagnoses. Indeed, the only study in the literature that included a clinical definition and a clinical setting for GD, similar to ours, was that by Heylens et al.^12^ That study, which showed a higher concordance in MZ than in DZ twins, was a hybrid study including 20 previously published case reports and 31 twin pairs that had attended a gender clinic and had been clinically assessed for gender identity disorder (GID), the latter being more comparable setting to our setting. The conclusion of higher concordance in MZ (39.1%) was heavily driven by the case reports, which are subject to publication bias. The 31 twin pairs that had a clinically assessed GID consisted of 6 MZ and 25 DZ pairs and the concordance rate in MZ pairs was 16.7% (1 out of 6 pairs) and 0% in DZ pairs. Most of the twins (N = 25) in that study came from a total of 561 children evaluated between 1976 and 2011 at the Gender Identity Service at the Centre for Addiction and Mental Health in Toronto, while three twin pairs came from the Gender Clinic of Ghent University Hospital during the period 1985–2010, from a pool of about 450 attendees and three twin pairs came from the Child and Adolescent Gender Clinic of Ghent University Hospital^12^. Interestingly, there were no concordant twins in this sample, including the 18 same-sex twin pairs (4 MZ and 14 DZ).

Moreover, 28 of the twin pairs the study by Heylens et al. were children, and 25 of those were younger than 12 at the diagnosis of GD. In our study, the mean age at diagnosis was 25 and 32 years for discordant and concordant twin pairs, respectively, which consists an interesting major difference between these studies. Even though the age at diagnosis is by no means the same with age of onset of GD, the vast majority of the twins included in the study by Heylens et al. had a clearly early onset of GD and a GD diagnosis in childhood, which was not the case in our study. Of note is that previous studies have stipulated that the effect of genetics on gender identity may be prone to changes during lifetime^14^. In particular, heritability may be a non-fixed parameter with an attenuated effect on gender identity throughout adolescence and adult life^14,23^.

Another study with a similar outcome was the study by Sasaki et al.^14^, that used self-assessment of GID with criteria based on DSM-IV. The primary outcomes of the study were prevalence and heritability of GID but the authors also reported on the concordance for GID among MZ, DZ, same- and different-sex twin pairs. That study showed a higher concordance among same-sex twin pairs (20.7%) than different-sex twin pairs (8.8%). Although the outcome was similar to ours, the study was population based and the authors recognize, among other potential limitations, the fact that their definition of GID does not necessarily reflect a clinical GID diagnosis.

Prenatal sex hormones have been considered to play a significant role in brain sexual differentiation through permanent organizing effects during a critical period of fetal brain organization, especially during the last trimester of the pregnancy^14,24–26^. These organizing structures are activated by sex hormones during puberty. Several studies have shown that sexually dimorphic brain structures seem to be more congruent with gender identity than the sex assigned at birth, possibly as a result of the effect of sex hormones, creating, thus, a restricted potential for change^27,28^. Indeed, our results of higher prevalence of GD in different-sex twins than that in same-sex twins and non-twin siblings in the present study, if replicated, provide some evidence that intrauterine factors, such as sex hormones produced by the co-twin, may influence the development of GD.

Some previous twin studies have provided evidence for a strong genetic influence in shaping gender identity. Candidate gene association studies have investigated whether functional variants in genes may alter sex hormone signaling, resulting in atypical sexual differentiation of the developing brains of those who will later experience GD^29,30^.

However, most studies have not used a stringent definition of GD as used in the present cohort, which may explain the null effect of heritability found herein. Indeed, the potential effect of genetics, if any, may apply to the development of gender identity as a trait, but not necessarily to the clinical diagnosis of GD. Future studies need to clarify the intricate interaction between genetics and environmental influences on gender identity and GD^26^.

### Strengths and limitations

In the present study we used, for the first time, registry data from a large Swedish population-based cohort over a long period (2001–2016) to study the familial aggregation of GD in the clinical setting, using a validated definition of GD. Our study with 67 twin pairs is larger than the one by Heylens et al., which is the largest available study with similar design studying clinical GD and which included 31 twin pairs. However, both studies are small and subject to random error. Moreover, the small power of the present study did not allow further sub-analyses, e.g., by age, to be conducted. Another limitation of our study was the lack of information about zygosity. However, it would be safe to assume that among the 40 same-sex pairs, approximately half of them are MZ pairs^31^, as almost one third of all twins are MZ^32^. The distribution of twin pairs in our study, i.e., the ratio between same-sex (n = 40) and different-sex (n = 27) pairs, was 1.5:1, which is in line with that reported by the Swedish Twin Registry^31^. The observation time from the first GD diagnosis varied from 1 to 15 years and thus we cannot exclude the possibility that some siblings and co-twins with shorter follow-up time could receive a GD diagnosis after the end of this study period and thus be misclassified as discordant. Lastly, the main outcome of this study was a clinically significant GD diagnosis according to our definition; thus, the present findings cannot provide assumptions on the whole picture of gender identity.

## Conclusions

The present register-based study aimed to examine the concordance for GD diagnosis utilizing a cohort of twin and non-twin sibling pairs in Sweden. We found no concordance among same-sex twins, as well as higher concordance among different-sex twin than non-twin sibling pairs. Beyond any potential limitations and biases, our findings, if replicated, suggest that familial factors, mainly confined to environmental influences during the intrauterine period, are more likely to explain the development of GD. Future studies are deemed necessary to shed light into the intricate interaction between genetic and environmental factors, as well as into the potentially relevant molecular pathways that underlie these associations, if causal.

## Data Availability

Our study includes data from Swedish health care registers, which cannot be shared due to confidentiality issues. Data are available from the National Board of Health and Welfare in Sweden (registerservice@socialstyrelsen.se) and Statistics Sweden (https://www.scb.se/om-scb/kontakta-oss/statistikservice/fraga-oss).
